# Supplementary material for: The efficacy of inspiratory muscle training in patients with coronary artery disease: Protocol for a systematic review and meta-analysis
Source: PLoS One. 2023 Sep 8;18(9):e0289287. doi: 10.1371/journal.pone.0289287 (PMC10490982; doi:10.1371/journal.pone.0289287)
Supplement: S2 Appendix — (DOCX) [file pone.0289287.s002.docx]

Appendix 2: search strategy

MEDLINE (Ovid) search strategy

| 1 | exp Inspiratory Capacity/ |
| --- | --- |
| 2 | Breathing Exercises/ |
| 3 | exp Respiratory Muscles/ |
| 4 | Inhalation/ |
| 5 | Respiratory Therapy/ |
| 6 | (inspiratory adj3 (condition* or endur* or exercis* or function* or flow or muscl* or pressur* or retrain* or re-train* or resist* or strength* or therap* or train* or weakness*)).ti,ab,kf. |
| 7 | ((inhal* or respiratory or breathing) adj2 (endur* or exercis* or muscl* or retrain* or re-train* or resist* or strength* or therap* or train* or weakness*)).ti,ab,kf. |
| 8 | (threshold adj2 (load or device*)).ti,ab,kf. |
| 9 | (resist* adj2 breath*).ti,ab,kf. |
| 10 | or/1-9 |
| 11 | exp Myocardial Ischemia/ |
| 12 | ((coronary adj2 (arter* or stenos* or atheroscleros* or arterioscleros*)) or (coronary adj5 disease?) or CAD).ti,ab,kf. |
| 13 | (angina or stroke?).ti,ab,kf. |
| 14 | ((heart or myocardial) adj3 infarct*).ti,ab,kf. |
| 15 | (isch?emi* adj3 (heart or cardiac or myocardial)).ti,ab,kf. |
| 16 | exp Myocardial Revascularization/ |
| 17 | (((aortocoronary or coronary) adj3 bypass*) or CABG).ti,ab. |
| 18 | (angioplast* or atherectom* or endarterectom* or thrombectom* or PCI or PTCA or (Percutaneous adj3 (intervent* or revascular*))).ti,ab,kf. |
| 19 | or/11-18 |
| 20 | 10 and 19 |
| 21 | randomized controlled trial.pt. |
| 22 | controlled clinical trial.pt. |
| 23 | randomized.ab. |
| 24 | placebo.ab. |
| 25 | drug therapy.fs. |
| 26 | randomly.ab. |
| 27 | trial.ab. |
| 28 | groups.ab. |
| 29 | 21 or 22 or 23 or 24 or 25 or 26 or 27 or 28 *(Cochrane Medline Sensitive RCT filter)* |
| 30 | exp animals/ not humans.sh. |
| 31 | 29 not 30 |
| 32 | 20 and 31 |

EMBASE (Ovid) search strategy

| 1 | inspiratory capacity/ |
| --- | --- |
| 2 | exp breathing exercise/ |
| 3 | breathing muscle/ |
| 4 | inhalation/ |
| 5 | respiratory care/ |
| 6 | (inspiratory adj3 (condition* or endur* or exercis* or function* or flow or muscl* or pressur* or retrain* or re-train* or resist* or strength* or therap* or train* or weakness*)).ti,ab,kw. |
| 7 | ((inhal* or respiratory or breathing) adj2 (endur* or exercis* or muscl* or retrain* or re-train* or resist* or strength* or therap* or train* or weakness*)).ti,ab,kw. |
| 8 | (threshold adj2 (load or device*)).ti,ab,kw. |
| 9 | (resist* adj2 breath*).ti,ab,kw. |
| 10 | or/1-9 |
| 11 | exp ischemic heart disease/ |
| 12 | ((coronary adj2 (arter* or stenos* or atheroscleros* or arterioscleros*)) or (coronary adj5 disease?) or CAD).ti,ab,kw. |
| 13 | (angina or stroke?).ti,ab,kw. |
| 14 | ((heart or myocardial) adj3 infarct*).ti,ab,kw. |
| 15 | (isch?emi* adj3 (heart or cardiac or myocardial)).ti,ab,kw. |
| 16 | exp coronary artery surgery/ |
| 17 | (((aortocoronary or coronary) adj3 bypass*) or CABG).ti,ab,kw. |
| 18 | (angioplast* or atherectom* or endarterectom* or thrombectom* or PCI or PTCA or (Percutaneous adj3 (intervent* or revascular*))).ti,ab,kw. |
| 19 | or/11-18 |
| 20 | 10 and 19 |
| 21 | crossover procedure/ |
| 22 | double blind procedure/ |
| 23 | exp randomized controlled trial/ |
| 24 | single blind procedure/ |
| 25 | (random$ or factorial$ or crossover$ or cross over$ or cross-over$ or placebo$ or (doubl$ adj blind$) or (singl$ adj blind$) or assign$ or allocat$ or volunteer$).mp. |
| 26 | 21 or 22 or 23 or 24 or 25 *(Cochrane Embase RCT filter)* |
| 27 | exp animal/ |
| 28 | exp human/ |
| 29 | 27 not 28 |
| 30 | 26 not 29 |
| 31 | 20 and 30 |

Cochrane Central Register of Controlled Trials (Ovid) search strategy

| 1 | exp Inspiratory Capacity/ |
| --- | --- |
| 2 | Breathing Exercises/ |
| 3 | exp Respiratory Muscles/ |
| 4 | Inhalation/ |
| 5 | Respiratory Therapy/ |
| 6 | (inspiratory adj3 (condition* or endur* or exercis* or function* or flow or muscl* or pressur* or retrain* or re-train* or resist* or strength* or therap* or train* or weakness*)).ti,ab. |
| 7 | ((inhal* or respiratory or breathing) adj2 (endur* or exercis* or muscl* or retrain* or re-train* or resist* or strength* or therap* or train* or weakness*)).ti,ab. |
| 8 | (threshold adj2 (load or device*)).ti,ab. |
| 9 | (resist* adj2 breath*).ti,ab. |
| 10 | or/1-9 |
| 11 | exp Myocardial Ischemia/ |
| 12 | ((coronary adj2 (arter* or stenos* or atheroscleros* or arterioscleros*)) or (coronary adj5 disease?) or CAD).ti,ab. |
| 13 | (angina or stroke?).ti,ab. |
| 14 | ((heart or myocardial) adj3 infarct*).ti,ab. |
| 15 | (isch?emi* adj3 (heart or cardiac or myocardial)).ti,ab. |
| 16 | exp Myocardial Revascularization/ |
| 17 | (((aortocoronary or coronary) adj3 bypass*) or CABG).ti,ab. |
| 18 | (angioplast* or atherectom* or endarterectom* or thrombectom* or PCI or PTCA or (Percutaneous adj3 (intervent* or revascular*))).ti,ab. |
| 19 | or/11-18 |
| 20 | 10 and 19 |

**PEDro search strategy**

1. (coronary OR ischemi* OR infarct* OR atheroscleros* OR CABG OR percutaneous OR PCI) AND (inspiratory OR inhal* OR respiratory OR breathing) [Abstract & Title]

2. clinical trial [Method]

3. 1 AND 2

**WHO ICTRP search strategy**

Condition: coronary OR myocardial OR ischem* OR ischaem* OR atherosclero* OR heart failure OR percutaneous

Intervention:

inspiratory OR inhal* OR respiratory

**ClinicalTrials.gov search strategy**

Study type: Interventional Studies (Clinical Trials)

Study Results: All Studies

Condition or disease: Coronary Artery Disease

Intervention/treatment: inspiratory OR breath* OR inhal* OR respiratory
